# Prevalence and phenotypic characterization of *Enterococcus species* isolated from clinical samples of pediatric patients in Jimma University Specialized Hospital, south west Ethiopia

**DOI:** 10.1186/s13104-018-3382-x

**Published:** 2018-05-08

**Authors:** Milkiyas Toru, Getnet Beyene, Tesfaye Kassa, Zeleke Gizachew, Rawleigh Howe, Biruk Yeshitila

**Affiliations:** 1 Dubo St General Hospital, Areka, Ethiopia; 2 School of Medical Laboratory Sciences, Institute of Health Sciences, Jimma University, POBox 378, Jimma, Ethiopia; 3 Arbaminch College of Health Sciences, Arbaminch, Ethiopia; 4 Armauer Hansen Research Institute, Addis Ababa, Ethiopia

**Keywords:** Prevalence, *Enterococcus* species, Virulence factor, Vancomycin, Southwest Ethiopia

## Abstract

**Objective:**

This study was done to determine the prevalence and phenotypic characterization of *Enterococcus* species isolated from clinical samples of pediatric patients in Jimma University Specialized Hospital, Southwest Ethiopia.

**Results:**

The overall prevalence of *Enterococci* species was 5.5% (22/403). Five (22.7%) of *Enterococci species* were vancomycin resistant. Haemolysin, gelatinase and biofilm production was seen among 45.5, 68.2 and 77.3% of isolates respectively. The overall rate of antibiotic resistance was 95.5% (21/22). High resistance was observed against norfloxacin (87.5%), and tetracycline (77.3%). Whereas, low resistance (36.5%) was observed against ciprofloxacin and eighteen (80.8%) of the isolates were multi-drug resistant.

**Electronic supplementary material:**

The online version of this article (10.1186/s13104-018-3382-x) contains supplementary material, which is available to authorized users.

## Introduction

*Enterococci* species are Gram-positive cocci that are normal inhabitants of gastrointestinal tract, oral cavity and female genital tracts in both humans and animals. However, they can also be significant pathogens responsible for serious nosocomial and community acquired infections, causing surgical wound infection, bacteraemia, endocarditis, neonatal sepsis and rarely meningitis [1–3].

Several virulence and pathogenicity factors have been described from the genus *Enterococcus* that enhances their ability to colonize human tissues, increase resistance to antibiotics, and aggravate infection outcomes [4, 5]. Moreover, the relative importance of the bacteria has increased with the occurrence of high-level resistance to multiple antimicrobials [6]. The emergence of vancomycin resistant *Enterococci* species (VRE) has alarmed the global community due to few options left for disease management. There is also a possibility for a resistance gene to transfer horizontally and increased virulence factors [7–9].

The prevalence of clinically isolated *Enterococcus*, especially VRE, was reported in Europe (4%), Asia–Pacific (11.9%), America (35.5%) and Latin America (12.9%) [10]. Increasing prevalence of VRE was also reported from few studies conducted in Ethiopia [11–13]. To our knowledge, there was no published data available on the epidemiology of *Enterococci* species infections among pediatric patients in Ethiopia. Therefore, this study attempted to assess the prevalence, antimicrobial susceptibility and virulence factors of *Enterococcus* species isolated from clinical samples of pediatric patients in Jimma University Specialized Hospital.

## Main text

### Materials and methods

#### Study area and period

The study was conducted at Jimma University Specialized Hospital (JUSH) in Jimma town which is located southwest of Addis Ababa, Ethiopia. JUSH provides services for approximately 17,500 inpatients and 164,000 outpatients/year. This hospital based study was undertaken from April to September, 2016.

#### Study design and study subjects

Hospital based cross sectional study design was employed. Pediatric patients younger than 15 years old were included in this study. Family/guardians gave written consent. Clinical specimens were collected and processed at Microbiology Laboratory of the hospital. Pediatric patients who received antibiotics within the past 2 weeks were excluded.

The sample size for the study was determined by using single population proportion formula considering the prevalence of *Enterococcus* species among pediatric patients as fifty percent (P = 50%). Accordingly, a total of 403 children were included in this study.

### Data collection

#### Socio-demographic and clinical data

Data on socio-demographic and clinical profile were collected. For this purpose semi-structured questioner was used for interview (history of chronic illness, hospitalization etc.,) and to extract participant’s medical record (sex, age, ward etc.,) [12, 13].

#### Laboratory data collection

A total of 403 different clinical specimens (urine, blood, swabs, closed abscess, body fluids and CSF) were collected from pediatric outpatient and inpatient departments following standard procedures [14].

#### Isolation and identification of *Enterococcus* species

Specimens were inoculated on appropriate culture media to isolate the bacteria. Briefly: The specimens were inoculated on Blood agar (Oxoid; Hampshire UK) with 5% sheep blood and MacConkey agar (bioMe´rieux; France). All media were kept at 37 °C for 24 h and Colonies larger than those of streptococci, usually 1–2 mm, with α, β or γ hemolysis on Blood agar or small dark-red magenta colonies on MacConkey agar were presumptively considered as *Enterococcus* species. Further identification was done with Gram stain, catalase production, esculin hydrolysis, salt tolerance (ability to grow in 6.5% NaCl broth) and l-pyrrolidonyl-b-naphthylamide (PYR) testing [14]. *Enterococcus faecalis* ATCC 29212 was used as positive control.

#### Antimicrobial susceptibility testing

This test was carried out using disk diffusion method on Muller Hinton agar (MHA) (Oxoid; Hampshire UK) according to the recommendation of Clinical and Laboratory Standard Institute (CLSI) of 2014 [15] for the following drugs: erythromycin (E, 10 µg), chloramphenicol (C, 30 µg), tetracycline (TE, 30 µg), ampicillin (AMP, 10 μg), norfloxacin (NOR, 30 μg), ciprofloxacin (CIP, 5 μg), penicillin (P, 10 IU) and vancomycin (VA, 30 μg) ‘(Oxoid, Hampshire England). These antimicrobial agents were selected based on the availability and prescription practices in JUSH and recommendations from CLSI, 2014 [15]. *Staphylococcus aureus* ATCC 25923 was used as a control strain.

#### Detection of virulence factors

Presence of beta-hemolysis surrounding *Enterococcus species* colonies after 24-h on blood agar incubated aerobically at 37 °C, was considered indicative of hemolysin production [16]. Gelatinase production was confirmed by observation of liquefaction into tubes containing 4 mL of nutrient broth (Himedia; INDIA) with 12% gelatine (Difco; USA) [5]. Biofilm formation *was* assessed using 96-wells flat bottom microtiter plates (Becton–Dickinson; Falcon USA) following the methods adopted by Banerjee et al. and Deka [16, 17].

#### Data processing and analysis

Data was entered, coded and cleaned in Epi Data version 3.1 software. Then it was exported to SPSS software (version 16.0) for analysis. Descriptive statistics was used to summarize socio-demographic, clinical profiles of the study participants and susceptibility patterns of the isolates. The findings were presented in tables. Chi square test was used to measure associations between variables. At 95% confidence intervals, P-values of less than 0.05 were considered as significant.

### Results

#### Socio-demographic characteristics of the study participants

A total of 403 children with age ranging from 0 to 14 years were included in this study. One clinical specimen was collected from each participant. More than half 53.1% (214/403) of the participants were female. The median age of study participant was 9 years. The age category distributions showed that the highest number of participants, 48.6% (196/403), were in the age group of 10–14 years. About 56.6% (228/403) of study participants were from rural setting and 54.1% (218/403) were not attending school. About 38.0% (153/403) of children’s parent/guardian had high school education and 28.3% (114/403) were farmers by occupation (Table 1).

**Table 1:** Socio-demographic and clinical characteristics of study participants at Jimma University Specialized Hospital, Southwest Ethiopia, April to September, 2016

Characteristics of study participants (n = 403) No. (%) of study participant	
Age in year	
0–4	121(30.0%)
5–9	86 (21.3%)
10–14	196 (48.6%)
Sex	
Male	185 (45.9%)
Female	218 (54.1%)
Place of residence	
Urban	175 (43.4%)
Rural	228 (56.6%)
Attending kindergarten/school	
Yes	185 (45.0%)
No	218 (54.1%)
Parent/guardian educational level	
College and above	105 (26.1%)
High school	153 (38.0%)
Primary school	67 (16.6%)
Read and write	40 (9.9%)
Cannot read and write	38 (9.4%)
Parents/guardian occupation	
Employed	88 (21.8%)
Merchant	79 (19.6%)
Farmer	114 (28.3%)
Housewife	104 (25.8%)
Others^a^	18 (4.5%)
Department	
OPD	157 (39%)
IPD	246 (61%)
Length of current hospitalization	
Not hospitalized	188 (46.7%)
< 2 weeks	203 (50.4%)
≥ 2 weeks	12 (3.0%)
Previous history of hospitalization	
Yes	32 (7.9%)
No	371 (92.1%)
History of invasive procedure	
Yes	44 (10.9%)
No	359 (89.1%)
History of antibiotic use	
Yes	132 (32.8%)
No	271 (67.2%)
Chronic illness	
Yes	37 (9.2%)
No	366 (90.8%)

^a^Student, daily labourer; OPD, outpatient department; IPD, in patient department

The clinical data showed that 53.2% (215/403) of the participants were hospitalized during the study time and 7.9% (32/403), 10.9% (44/403) and 32.8% (132/403) had previous history of hospitalization, invasive procedure and antibiotic use respectively. About 9.2% (37/403) of the children had confirmed chronic illness (Table 1).

#### Risk Factors for Vancomycin resistant *Enterococcus* species infection

Prevalence of VRE infection was significantly associated with current length of hospitalization for ≥ 2 weeks (P = 0.025). But there was no statistically significant difference (*P* value > 0.05) with hospital department, length of previous hospitalization, history of invasive procedure, history of antibiotic use, types of invasive procedure and history of chronic illness (Additional file 1).

#### Prevalence of *Enterococcus* species and virulence factors

The overall prevalence of *Enterococcus* species was 5.5% (22/403). The majority (19/22) of isolated *Enterococcus* species were positive for production of at least one of three virulence factors (Haemolysin, Gelatinase and Biofilm) (Table 2). Furthermore, 77.3, 45.5 and 68.2% of the isolates were positive for biofilm, haemolysin and gelatinase production respectively (Table 2). Seven (31.8%) strains were positive for all the three virulence properties whereas none of the virulence factors were positive for three Enterococcus isolates.

**Table 2:** Detection of virulence factors in *Enterococcus* species isolates

Virulence factor	Haemolysin	Gelatinase	Biofilms	Total no. (%)^a^
Combination of factors	+	+	+	7 (31.8%)
	+	+	−	0 (0.0%)
	+	−	+	1 (4.5%)
	−	+	+	8 (36.4%)
	+	−	−	2 (9.1%)
	−	+	−	0 (0.0%)
	−	−	+	1 (4.5%)
	−	−	−	3 (13.6%)
Total (%)	10 (45.5%)	15 (68.2%)	17 (77.3%)	22 (100.0%)

^a^Total number of isolates which produce virulence factors

#### Antimicrobial susceptibility patterns of isolates

The overall rate of resistance was 95.5% (21/22). High rate of resistance was observed against norfloxacin (87.5%) and tetracycline, whereas, low rate of resistance was observed against ciprofloxacin (36.4%) and vancomycin (22.7%) (Table 3).

**Table 3:** Antimicrobial resistance patterns of *Enterococcus* species isolated from clinical samples of pediatric patients in Jimma University Specialized Hospital, April to September, 2016

Antimicrobial agents	No. (%) of resistant isolates
Ampicillin	12 (54.5)
Penicillin	14 (63.6)
Vancomycin	5 (22.7)
Erythromycin	14 (63.6)
Tetracycline	17 (77.3)
Ciprofloxacin	8 (36.4)
Chloramphenicol	14 (63.6)
Norfloxacin^a^	7 (87.5)^a^

No significant association was found between virulence factors and resistance pattern of the isolated *Enterococci* species to an antibiotic (Y > 0.05) (Additional file 2)

^a^Tested only for urinary isolates (n = 8)

### Discussion

In our study, the overall prevalence of *Enterococcus* species was 5.5%. This result was comparable with studies reported that ranges between 5.0 and 6.2% [18–21]. The rate in our study is lower than 11.0% reported from Malaysia [22], 20.8% from Pakistan [23] and 15.3% from Tanzania [24], but higher than 3.2% in Istanbul, Turkey [25], 1.4% in São Paulo, [26] and 2.7% in Port Sudan in all age group [27]. These differences in prevalence might be due to methodological design used (retrospective and cohort), study area and study period in previous studies.

In our study, length of current hospitalization for ≥ 2 weeks was significantly important factor (P = 0.025). Our study finding is concordant with most previous studies from Egypt [28]. Longer hospital stays can indicate a greater chance of receiving antibiotics and also a longer exposure time to possible pathogen selection or transmission.

The current study showed that 22.7% of the isolates were resistant to vancomycin which is comparable with finding from Tabriz in Iran [29] and Mansoura in Egypt [28], but higher than the prevalence reported from Turkey, 1.55% [30]; 10% [31] and 16% [32] from Iran. But lower than the findings from Serbia, 54% [33]; Iran [34] and Nigeria, 42.9% [20] in clinical isolates from all age groups. The increased prevalence of VRE infection in our study might be associated with the availability and use of vancomycin in Jimma Specialized Hospital.

In our study, penicillin and ampicillin resistance was seen in 63.6 and 54.5% of Enterococcal isolates, respectively; which is relatively comparable with study result reported from China in all age group [35] and Jimma, Ethiopia [12]. On the other hand, our finding is lower than findings from India [36, 37]. In our study 63.6% of the isolates were resistant against erythromycin which is similar with 67.0% reported in Port Sudan from all age group [27] and 63.2% from both Gondar and Jimma, Ethiopia in stool isolates from adults in separate study [12, 13]. However, lower than reported from Osogbo, Nigeria from all age group [20] and in Dilla, Ethiopia [11] but, higher than study findings from Iran [34], India [37] and China [35].

Higher rate of resistance to chloramphenicol (63.6%) was also observed which is comparable with study from Tanzania [24] and much higher than resistance rate reported from Gondar, 12.4% [13] and Dilla, 3.8% [11] in Ethiopia. In this study, lower rate of resistance was seen against ciprofloxacin (36.4%) which is comparable with study reported in Sudan [27] and India [38] and in Gondar, Ethiopia where the rate was 33.8% among isolates from stool specimens [13]. Resistance rate may go parallel with frequent use and availability of the drug in the local health institutions in the past times.

Among the total of the Enterococcal isolates, 68.2% were gelatinase producer which is in agreement with study reported from Skopje, Macedonia [39] and Porto Alegre, Brazil [40] and higher than 3.7% in Dilla, Ethiopia from faecal isolates [11] and lower than compared to 84% in Cairo, Egypt from clinical isolates in all age groups [41]. This variation might be due to difference in distribution of virulent strains in different geographical areas. Haemolysin was produced by 45.5% of the *Enterococci* species isolate which is comparable with the result of a study conducted in Skopje, Macedonia where 50% of the isolates produces haemolysin [39]. However, it is higher than those reported from Varanasi, North India and South India with 36.6 and 16.5%, respectively [16, 42]; and from Norway, 17% [43] and very low compared to 2% from Dilla, Ethiopia for isolates from faecal sources [11].

In this study, 77.3% of the isolates were biofilm formers. This is similar with previous report from Iraq, 77.3% [44] and comparable to Porto Alegre, Brazil, 74% [40] and higher than 26.12% in North India [16], 32.5% in Bangalore, India [42], and 64.4% in Dhaka [9]. The differences in biofilm formation might be due to strain variation. Unlike report from Dhaka [9] and India [16] where there was strong association between virulent factors production and antibiotic resistance, the findings of our study showed no association between them, however, all biofilm formers and gelatinase producers showed high resistance.

### Conclusion

The finding of this study indicated the presence of *Enterococci* species with different virulence factors. *Enterococcus* species have shown an increased rate of resistance to most tested drugs particularly to vancomycin. This finding demands an attention from health policy makers for intensified actions to promote rational use of antibiotics in health care settings and surveillance studies in order to monitor changes in enterococcal resistance patterns.

## Limitations of the study

Because of lack of API 20 strep strip or biochemical reagents for species identification, we were unable to detect different species of *Enterococci* species and could not see the association of virulent factors as well as antimicrobial resistance profile against the type of isolated species.

Vancomycin resistance was determined using disk diffusion method.

## Additional files

**Additional file 1.** Socio Demographic and clinical characteristics of pediatric patients infected with Vancomycin Resistant Enterococci species (VRE) and Vancomycin sensitive Enterococci species (VSE) at Jimma University Specialized hospital, April to September, 2016.

**Additional file 2.** Association between virulence factors and antimicrobial resistance of *Enterococcus* species isolated from clinical samples of pediatric patients.

## Abbreviations

VRE: vancomycin resistant *Enterococci* species
HIV: human immunodeficiency virus
JUSH: Jimma University Specialized Hospital
