# Correction to: Prevalence and phenotypic characterization of *Enterococcus species* isolated from clinical samples of pediatric patients in Jimma University Specialized Hospital, south west Ethiopia

**DOI:** 10.1186/s13104-019-4290-4

**Published:** 2019-05-07

**Authors:** Milkiyas Toru, Getnet Beyene, Tesfaye Kassa, Zeleke Gizachew, Rawleigh Howe, Biruk Yeshitela

**Affiliations:** 1 Dubo St General Hospital, Areka, Ethiopia; 2 School of Medical Laboratory Sciences, Institute of Health Sciences, Jimma University, POBox 378, Jimma, Ethiopia; 3 Arbaminch College of Health Sciences, Arbaminch, Ethiopia; 4 Armauer Hansen Research Institute, Addis Ababa, Ethiopia

## Correction to: BMC Res Notes (2018) 11:281 10.1186/s13104-018-3382-x

Following publication of the original article [1], the authors reported that one of the authors’ names was spelled incorrectly. In this Correction the incorrect and correct author name are shown.

Originally the author name was published as:

- Biruk Yeshitila

The correct author name is:

- Biruk Yeshitela
