# Supplementary material for: Prevalence and phenotypic characterization of Enterococcus species isolated from clinical samples of pediatric patients in Jimma University Specialized Hospital, south west Ethiopia
Source: BMC Res Notes. 2018 May 8;11:281. doi: 10.1186/s13104-018-3382-x (PMC5941600; doi:10.1186/s13104-018-3382-x)
Supplement: Supplementary file 1 — Additional file 1. Socio Demographic and clinical characteristics of pediatric patients infected with Vancomycin Resistant Enterococci species (VRE) and Vancomycin sensitive Enterococci species (VSE) at Jimma University Specialized hospital, April to September, 2016. [file 13104_2018_3382_MOESM1_ESM.docx]

**Additional file 1:** Socio Demographic and clinical characteristics of pediatric patients infected with Vancomycin Resistant *Enterococci* species (VRE) and Vancomycin sensitive *Enterococci* species (VSE) at Jimma University Specialized hospital, April to September, 2016.

| Variables | *Enterococcus species* (n=22) | | | X^2^- test  P-value |
| --- | --- | --- | --- | --- |
|  | **VRE (n=5) VSE (n=17)** | | |  |
|  | No. (%) No. (%) | | |  |
| **Sex**  Female  Male | 3(23.1)  2(22.2) | | 10(76.9)  7(77.8) | 1.000 |
| **Age in years** | | | | |
| 0-4  5-9  10-14 | | 1(25.0)  1(33.3)  3(20.0) | 3(75.0)  2(66.7)  12(80.0) | 0.751 |
| **Residence** | | |  |  |
| Rural  Urban | | 4(44.4)  1(7.7) | 5(55.6)  12(92.3) | 1.000 |
| **Department** | | |  |  |
| OPD  IPD | | 1(12.5)  4(28.6) | 7(87.5)  10(71.4) | 0.736 |
| **Ward** | | |  |  |
| Pediatrics  Surgical  ICU  Others | | 0(0.0)  2(33.3)  1(25.0)  1(50.0) | 2(100)  4(66.7)  3(75.0)  1(50.0) | 1.000 |
| **Length of current hospitalization** | | |  |  |
| Not hosp.  <2weeks  ≥2weeks | | 1(11.1)  0(0.0)  4(66.7) | 8(88.9)  7(100.0)  2(33.3) | **0.025** |
| **Previous history of hospitalization** 3(23.1) | | | 10(76.9) | 1.000 |
| **History of Invasive procedure** 5(26.3) | | | 14(73.7) | 0.788 |
|  | | |  |  |
| **History of antibiotic use** 4(57.1) | | | 15(42.9) | 1.000 |
| **Chronic illness** 3(27.3) | | | 8(72.7) | 1.000 |

**VRE, Vancomycin Resistant *Enterococci* species;** **VSE,** **Vancomycin sensitive *Enterococci* species;** **OPD, outpatient department; IPD, inpatient department;** **ICU, intensive care unit**
