# Supplementary material for: Prevalence and phenotypic characterization of Enterococcus species isolated from clinical samples of pediatric patients in Jimma University Specialized Hospital, south west Ethiopia
Source: BMC Res Notes. 2018 May 8;11:281. doi: 10.1186/s13104-018-3382-x (PMC5941600; doi:10.1186/s13104-018-3382-x)
Supplement: Supplementary file 2 — Additional file 2. Association between virulence factors and antimicrobial resistance of Enterococcus species isolated from clinical samples of pediatric patients. [file 13104_2018_3382_MOESM2_ESM.docx]

Additional file 2: Association between virulence factors and antimicrobial resistance of *Enterococcus species* isolated from clinical samples of pediatric patients.

| Resistant antibiotics | Biofilm formation | | | Gelatinase production | | | Haemolysin production | | |
| --- | --- | --- | --- | --- | --- | --- | --- | --- | --- |
|  | Former (n=17) | NF^$^ (n=5) | P- value | Producer ( n=15) | NP^*^ (n=7) | P- value | Producer (n=10) | NP^*^ (n=12) | p- value |
|  | No. (%) | No.(%) |  | No. (%) | No.(%) |  | No. (%) | No.(%) |  |
| Vancomycin | 4(23.5) | 1(20) | 1.000 | 3(20) | 2(28.6) | 1.000 | 3(30) | 2(16.7) | 0.816 |
| Ampicillin | 10(58.8) | 2(40) | 0.816 | 8(53.3) | 4(57.1) | 1.000 | 6(60) | 6(50) | 0.969 |
| Penicillin | 12(70.6) | 2(40) | 0.471 | 11(73.3) | 3(42.8) | 0.364 | 7(70) | 7(58.3) | 0.903 |
| Erythromycin | 11(64.7) | 3(60) | 1.000 | 9(60) | 5(71.4) | 0.966 | 6(60) | 8(66.7) | 1.000 |
| Tetracycline | 13(76.5) | 4(80) | 1.000 | 12(80) | 5(71.4) | 1.000 | 8(80) | 9(75) | 1.000 |
| Ciprofloxacin | 7(41.2) | 1(20) | 0.736 | 7(46.6) | 1(14.2) | 0.320 | 6(60) | 2(16.7) | 0.097 |
| Chloramph.^a^ | 13(76.5) | 1(20) | 0.075 | 11(73.3) | 3(42.8) | 0.364 | 8(80) | 6(50) | 0.312 |
| Norfloxacin | 7(41.2) | 0(0.0) | 0.225 | 7(46.6) | 0(0.0) | 0.225 | 3(30) | 4(33.3) | 1.000 |
| ^$^NF, non-formers; ^*^NP, non-producers; ^a^Chloramph., Chloramphenicol. | | | | | | | | | |
